# The role of salvage surgery with interstitial brachytherapy for the Management of Regionally Recurrent Head and Neck Cancers

**DOI:** 10.1186/s41199-019-0043-2

**Published:** 2019-07-22

**Authors:** Nayel Khan, Mark Clemens, Jun Liu, Adam S. Garden, Anne Lawyer, Randal Weber, G. Brandon Gunn, William H. Morrison, Michael E. Kupferman

**Affiliations:** 1 Baylor College of Medicine, Houston, TX USA; 2 Department of Plastic Surgery, University of Texas M. D. Anderson Cancer Center, Houston, TX USA; 3 Department of Radiation Oncology, University of Texas M. D. Anderson Cancer Center, Houston, TX USA; 4 Department of Head and Neck Surgery, University of Texas M. D. Anderson Cancer Center, Unit 441, 1515 Holcombe Boulevard, Houston, TX 77096 USA

**Keywords:** Brachytherapy, Neck dissection, Recurrence, Squamous cell carcinoma

## Abstract

**Background:**

The optimal treatment for regional lymphatic recurrences from head and neck cancer has not been fully established. In order to explore the therapeutic benefit of surgical resection and adjuvant brachytherapy, the authors reviewed their experience utilizing interstitial brachytherapy (IBT) at the M. D. Anderson Cancer Center.

**Methods:**

A retrospective chart review of the 51 patients who received salvage surgical resection of lymphatic recurrences and adjuvant IBT between 1993 and 2012 at the M. D. Anderson Cancer Center was undertaken. All patients underwent neck dissection with complete resection and intraoperative placement of afterloading brachytherapy catheters. Soft tissue reconstruction was performed as necessary. The technical aspects of IBT were reviewed, and the overall and disease free survival rates and the recurrence rates were determined.

**Results:**

All patients had received external beam radiation (EBRT) as part of their initial treatment to a median dose of 66 Gy; 40 and 68% of the patients also had a neck dissection or chemotherapy, respectively. The cumulative regional recurrence probability is 28 and 38% at 5 years and 10 years. All of the patients underwent salvage neck dissection and IBT, with 81% also undergoing soft tissue reconstruction. The median dose delivered to the tumor bed was 60 Gy over a median duration of 4.5 days. There were 21 early adverse events, 8 of which were severe, and 19 late adverse events, 6 of which were severe. The most common early and late adverse events due to surgery and brachytherapy were dysphagia (7.1%) and true vocal cord paralysis (17.9%), respectively. There were no perioperative deaths or carotid hemorrhages. Nineteen patients developed recurrence including regional recurrence and distinct metastasis. The median time to recurrence is 130 months using Kaplan-Meier product limit method. The 2-year disease-free survival rate was 58%. The 2-year, 5-year, and 10-year overall survival rates were 69, 56, and 46%, respectively.

**Conclusions:**

Regional recurrences in previously irradiated tissues after the definitive treatment of primary head and neck cancers represent a challenging problem. We demonstrated that salvage neck dissection with IBT provided encouraging regional control and survival rates, while maintaining relatively low acute and long-term toxicity rates.

## Introduction

Head and neck squamous cell carcinoma (SCC) is the sixth most common type of cancer in the United States with an estimated 53,640 new cases and 11,520 deaths in 2013 [1]. Treatment options for these tumors include surgical resection, chemotherapy, and external beam radiation therapy (EBRT). Current management strategies include monotherapy (often resection or EBRT) for early stage disease and multimodality therapy for advanced stage disease. Despite aggressive treatment regimens, local and regional recurrences still frequently occur.

Regional recurrences are difficult to manage and pose challenging dilemmas for the physician. Pathologically, these recurrences commonly have unfavorable prognostic features such as extracapsular spread (ECS), often with extensive soft tissue involvement and perineural invasion (PNI). When the recurrences occur in previously irradiated regions, the risk of developing serious complications from re-irradiation is significant. For this reason, salvage treatment options are often limited to radical surgical resections when amenable (in as little as 35% in one study [2]) and/or palliative chemotherapy. These choices, however, are suboptimal, as single modality therapy has a low likelihood of success without adjuvant radiation therapy. Interstitial brachytherapy (IBT) has the advantage over external beam radiation of delivering its tumoricidal dose over a short treatment duration directly to the high-risk tumor bed while limiting unwanted dose to surrounding non-target tissues, such as overlying skin, neurovascular structures, and bone. We previously published our clinical experience using surgery with IBT for isolated neck recurrences [3].

The objective of this analysis was to assess the outcomes of salvage neck dissection in conjunction with IBT for the management of recurrent neck metastases in an expanded patient cohort with long-term follow up.

## Materials and methods

Between 1993 and 2012, 56 patients underwent cervical lymphadenectomy and adjuvant IBT with curative intent for the treatment of regional recurrences of head and neck malignancies at M. D. Anderson Cancer Center. A chart review of the patient population was performed after approval was obtained from the Institutional Review Board. Excluded patients included two who received lower dose brachytherapy intended to serve as a boost dose to external beam radiation, a foreign patient with less than 1 month follow up, a metastatic ovarian case; the other excluded patient had a radiation associated sarcoma that occurred 8 years after radiation in the supraclavicular field of a breast cancer patient. Fifty-one patients were included in this study. Data examined included demographic data, comorbidities, primary tumor characteristics, details of the initial treatment, details of the salvage treatment, and patient oncologic and toxicity outcomes.

All patients were evaluated preoperatively with a thorough history and physical examination, routine hematology and chemistries, axial imaging of the head and neck, and chest radiography. Pathologic diagnoses were confirmed with tissue biopsies.

IBT following complete surgical resection was pursued if the potential residual microscopic disease could be encompassed by a single-plane brachytherapy implant. Preferred candidates were those who had neck recurrence more than 5 months after completion of initial EBRT. Neck dissection was classified as selective neck, modified radical, or radical neck depending upon the lymphatic and non-lymphatic structures resected. Intraoperatively, after surgical resection and with the wound bed open, the radiation oncologist and surgeon placed the afterloading catheters sequentially through the skin flaps of the neck dissection and into the tumor bed at exact 1 cm intervals. The number of catheters inserted varied in order to ensure 1.5 to 2 cm margins around the tumor bed. The median number of catheters placed was 8 (range 5–14). The catheters were secured with several absorbable sutures. If the tumor was resected off the carotid artery, then the catheters were placed perpendicular to and directly across the carotid. Soft tissue reconstruction with a pedicled or free flap was performed as indicated to ensure wound healing. The estimated peripheral margins of the tumor bed were marked with surgical clips to facilitate postoperative dosimetry planning.

Within 48 h after surgery, patients underwent planning radiography, more recently with computed tomography, and a brachytherapy treatment plan was developed. Four to five days postoperatively, for the initial 45 patients, the catheters were loaded with ^192^Ir wire per individualized dose prescriptions dependent on the clinical scenario. The final eight patients were irradiated with pulse dose rate iridium methodology using a Nucleotron microSelectron. The implants were dosed at 5 mm from the plane of the sources with optimization of the treatment plan to ensure uniformity of dose and coverage of the tumor bed.

One radiation oncologist (WHM) performed the majority of the interstitial implants and supervised the head and neck brachytherapy program over the 19 years of this series, which ensured uniformity of doses. The median total dose was 60 Gy; 38 patients received this dose. Total dose ranged between 40 and 62 Gy. Two patients, who were implanted 2 months and 3.5 months after completing external beam radiation, received 40 and 45 Gy, respectively. Two patients, one with no extracapsular extension and the other with negative margins, were considered to be favorable presentations and were treated to a reduced dose of 55 Gy. Another patient received a lower dose of 55 Gy because his interstitial implant was close to the brachial plexus. The median dose-rate was 60 cGy per hour (range, 36 to 90 cGy/hr). The total milligram Radium equivalent (mgRaEq) per implant used varied between 12 and 66 for the iridium wire implants, with a median value of 34 mgRaEq. The median active total length of Iridium wire used was 58 cm (range 24 to 118 cm.) All patients were monitored postoperatively and during their inpatient brachytherapy treatment course for any surgical or radiation-based complications. All patients were prescribed a regimen of physical therapy for postoperative rehabilitation.

Patient outcomes were assessed, and the recurrence, disease-free survival, and overall survival rates were calculated via the Kaplan-Meier method. Adverse events were defined as either acute (occurring within the first 30 days postoperatively) or late (occurring more than 30 days postoperatively) and graded according to the Common Toxicity Criteria for Adverse Events (version 4.0) [4]. Pathology reports were reviewed for adverse pathologic features, including extracapsular spread, perineural invasion, and soft tissue involvement. Follow up period was determined by last encounter note or last correspondence with patient. The overall survival was defined as the time interval from surgery date to the death date or the last follow-up date, whichever occurred first. The disease free survival was defined as the time interval from surgery date to the recurrence, death or the last follow-up dates, whichever occurred first. Patients who were alive at the last follow-up were censored in the analyses. The cumulative survival rates were estimated by the Kaplan-Meier product-limit method. Statistical analysis was performed in SAS 9.4 (SAS Institute Inc., Cary, NC) and R (The R Foundation for Statistical Computing).

## Results

### Patient characteristics

We identified 51 patients who underwent salvage neck dissections followed by adjuvant IBT for regional recurrences. The basic demographic data of the patients and the key features of the initial primary cancers are described in Tables 1. The median follow up period was 40.7 months (range, 2.3–225 months).

**Table 1:** Patient Characteristics

Variable	Frequency (%)
Age median [range], years	57.6 [28.6–84.0]
Gender	
Male	39 (77%)
Female	12 (23%)
Significant history of tobacco use	
Yes	34 (67%)
No	17 (33%)
Significant history of alcohol use	
Yes	13 (25%)
No	38 (75%)
Primary Site	
Oropharynx	22 (43%)
Unknown Primary	7 (13%)
Hypopharynx	4 (8%)
Skin	4 (8%)
Larynx	4 (8%)
Major Salivary Gland	4 (8%)
Sinonasal	3 (6%)
Nasopharynx	2 (4%)
Oral cavity	1 (2%)
Pathology	
Squamous cell carcinoma	47 (92%)
Acinic cell carcinoma	1 (2%)
Adenoid cystic carcinoma	1 (2%)
Myoepithelial carcinoma	1 (2%)
Salivary duct carcinoma	1 (2%)
T stage	
2	2 (4%)
3	7 (14%)
4A	36 (70%)
4B	4 (8%)
4C	2 (4%)
Prior neck dissection	
Yes	27 (53%)
No	24 (47%)
Chemotherapy (initial treatment)	
Yes	36 (71%)
No	15 (29%)
Median initial XRT dose [range], Gy	66.0 [50–72]
Type of salvage Neck Dissection	
Modified Radical Neck Dissection	36 (71%)
Radical Neck Dissection	15 (29%)
Interstitial Brachytherapy	
Median dose [range], Gy	60 [40–62]
Average dose ± std., Gy	57.7 ± 4.8
Average duration [range], d	5 [3–6]
Average dose rate [range], cGy/h	60 (36–90)

XRT, radiation therapy; Gy, Gray; mo, months;

std., standard deviation; d, days; cGy/h, centiGray per hour;† mean time to neck recurrence is calculated for 14 patients who had local regional recurrence

### Initial treatments

The initial treatment of the patients’ primary tumors is summarized in Table 1. All patients had received EBRT as a component of their initial cancer care to a median dose of 66 Gy (range, 50–72 Gy). Twenty-seven (52.9%) patients underwent a neck dissection as part of the initial therapy, and 36 (70.6%) of the patients received chemotherapy.

### Salvage treatment

All of the patients underwent salvage neck dissection with intraoperative placement of afterloading catheters and postoperative dosimetry planning and brachytherapy delivery, as shown in Table 1. Soft tissue reconstruction was performed in 43 of the 51 patients (81%). Histopathologic review of tumor specimens obtained from the patients who underwent salvage resection and IBT for neck recurrences revealed that 50 (98%) and 14 (28.5%) had ECS and PNI, respectively (Table 2). The median IBT dose was 60 Gy (range, 40–62 Gy) delivered over an average duration of 5 days (range, 3–6) with a average dose-rate of 60 cGy/hour (range, 36–90 cGy/hour). All patients completed IBT as prescribed.

**Table 2:** Pathologic Findings at Salvage Surgery

Variable	Frequency (%)
Extracapsular Extension	
Yes	50 (98%)
No	1 (2%)
Perineural Invasion	
Yes	14 (28%)
No	37 (73%)
Margins	
Positive	5
Negative	9
Not Reported	37
Gross Residual Disease	1

### Outcomes

**Fig. 1:**
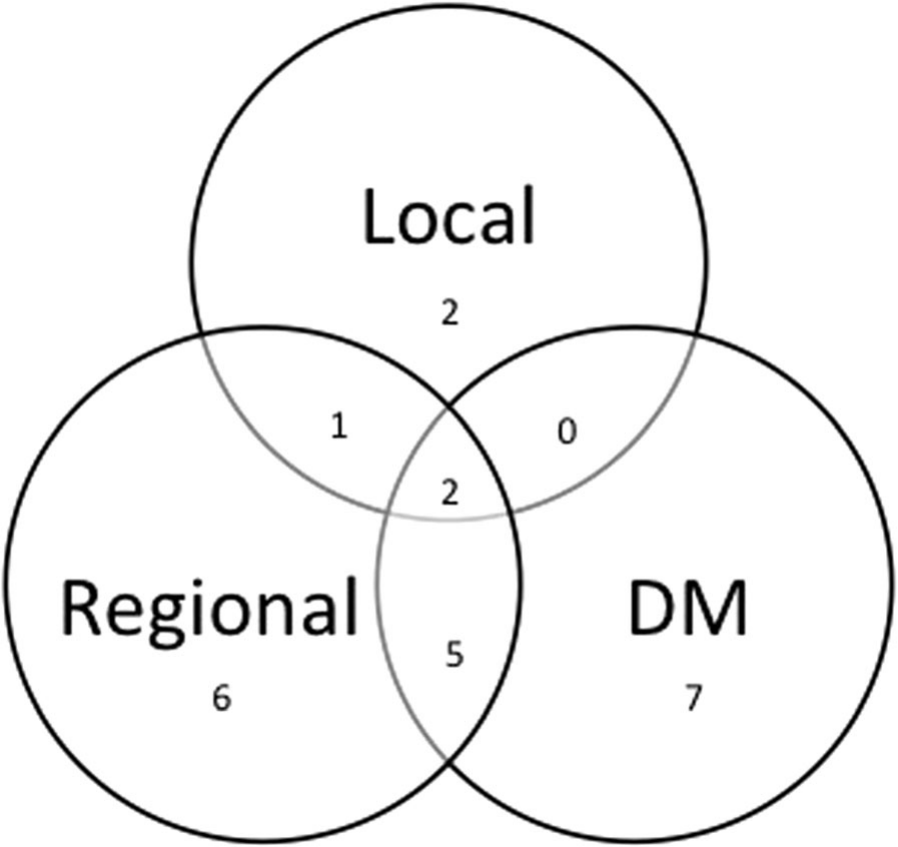
Patterns of failure after salvage neck dissection and IBT. DM indicates distant metastases. Numbers represent the number of affected patients

**Fig. 2:**
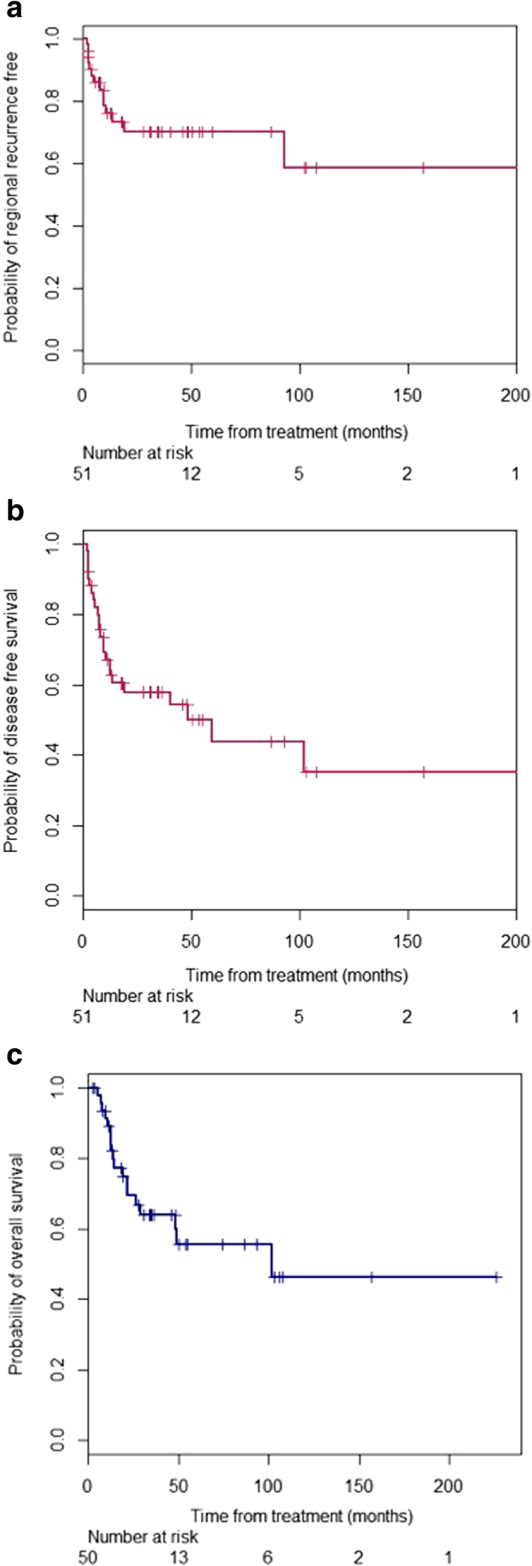
Kaplan-Meier curve of (**a**) disease-free survival, (**b**) overall survival for patients who underwent salvage neck dissection and IBT for cervical lymphatic recurrences and (**c**) probability of regional recurrence free after salvage IBT, including neck recurrences both inside and out of the brachytherapy treatment volume

Nineteen patients (37%) developed recurrences after salvage therapy and five patients died before recurrence developed. The pattern of recurrence is shown in Fig. 1. Fourteen patients had a regional recurrence after completing surgery and IBT. Six of these occurred in the IBT volume, while eight recurrences were located in the neck but outside of the IBT irradiated field. When taking account of censorship, the cumulative probability of regional recurrence is less than 50% at the last follow up so the median time to regional recurrence has not been reached (Fig. 2A). The cumulative regional recurrence probability is 28 and 38% at 5 years and 10 years. Six of 10 (60%) patients who were treated with IBT for progressive/recurrent disease less than 6 months after initial treatment developed a recurrence, while 13 of 41 (31.7%) patients who were treated for recurrent disease greater than 6 months after initial therapy had a recurrence. Nineteen patients developed recurrence including regional recurrence and distinct metastasis. The median time to recurrence is 130 months by using Kaplan-Meier product limit method. In total, 27 patients were alive without disease at the last follow up. The 2-year disease-free survival rate was 58% and the median time to recurrence/death is 59 months (Fig. 2B). Eighteen out of 51 patients died within follow up period. The median time to death was 101 months (95% CI = 28.8–225 months). The 2-year, 5-year, and 10-year overall survival rates were 69, 56, and 46%, respectively (Fig. 2C).

### Complications

Adverse outcomes as a result of the neck dissection and/or IBT are displayed in Table 3. Early adverse events were observed in 21 patients (39.6%) with 8 (15.1%) considered as grade 3 or 4 events. Four of the more severe events developed after salvage neck dissection but before brachytherapy had started. In one instance, the patient suffered from severe laryngeal edema soon after being extubated and required re-intubation. Another patient suffered from difficulty breathing and required the use of noninvasive positive pressure ventilation. Two patients developed complications pertaining to the soft tissue reconstruction.. There were no perioperative or treatment-related deaths.

**Table 3:** The Incidence of Early (< 30 Days) and Late (> 30 Days) Adverse Events After Salvage Neck Dissection and Interstitial Brachytherapy

	Acute toxicity			Late toxicity	
Grade	No. of patients	%	Grade	No. of patients	%
Grade 4		1.9	Grade 4		5.7
Edema, larynx	1		Soft tissue necrosis: Neck	3	
Grade 3		13.2	Grade 3		5.7
Dyspnea	1		Osteonecrosis	1	
Flap thrombosis	1		Soft tissue necrosis: Neck	2	
Hematoma	1				
Infection: Respiratory	2				
Infection: Skin, cellulitis	2				
Grade 2		13.2	Grade 2		24.5
Dysphagia	4		Horner syndrome	1	
Hematoma	1		Hypothyroidism	2	
Mucositis: Oral	1		TVC paralysis	10	
Postoperative delirium	1				
Grade 1		11.3	Grade 1		1.9
Chylothorax	1		Neuropathy: Motor	1	
Hypoglossal nerve dysfunction	1				
Muscle weakness: Facial	1				
Wound complication: Noninfectious	1				
Xerostomia	2				

No. indicates number; %, percent of sample; TVC, true vocal card

Late adverse events were observed in 19 patients (35.8%) with 6 (11.3%) considered as grade 3 or 4 events. Five of the six severe late adverse events were related to local wound complications. A pathologic fracture of the mandible occurred in a patient whose catheters were required to be placed directly under the mandible to ensure tumor bed coverage; this complication was successfully treated with a partial mandibulectomy. The most common late adverse event observed was unilateral true vocal cord paralysis, which occurred in 10 patients (17.9%). Vagal nerve injury was the probable cause of the vocal cord paralysis in these patients. The ipsilateral vocal cord in these patients on examination was located in the paramedian position and was paralyzed. Late carotid hemorrhage did not occur, even though several implants were placed directly across the carotid artery.

## Discussion

Recurrent regional disease of head and neck cancers poses a difficult problem, particularly among patients who have been previously treated with radiotherapy. While salvage surgery is often the mainstay of treatment, it is often ineffective without adjuvant radiotherapy. Studies have shown that salvage surgery has a 5-year disease-specific survival of 55–70% among patients treated with radiotherapy for laryngeal carcinoma [5]. However, salvage surgery in the setting of recurrent oropharyngeal SCC has a 5-year overall survival rate of only 28 to 32% [6, 7]. Furthermore, recurrent disease is often not amenable to surgical resection, with nearly 65% of patients with recurrent disease deemed inoperable due to location, extent, and patient comorbidities, among others [2]. In addition, in those instances where the disease is resectable, adverse prognostic characteristics such as ECS or PNI limit the effectiveness of single-modality therapy. Therefore, the optimal patient is one with an isolated or limited neck recurrence that can be adequately salvaged with complete surgical resection of gross tumor and local irradiation to eradicate residual microscopic disease.

Chemotherapy alone is generally not considered curative for these localized neck recurrences, and primary reirradiation with EBRT is associated with increased risk of serious normal tissue injury. Results from the Phase III multicenter Erbitux in First-Line Treatment of Recurrent or Metastatic Head and Neck Cancer (EXTREME) trial in patients with recurrent or metastatic head and neck SCC found that platinum-based chemotherapy protocols result in only a modest increase in overall survival (7.4 months), which is marginally improved with the addition of EGFR-inhibitors such as cetuximab (10.1 months) [8].

Because radiotherapy is often a component of initial treatment for upper aerodigestive tract cancers, re-irradiation of these areas to treat recurrences via EBRT carries increased risk. The prior radiotherapy narrows the therapeutic index and either limits the dosage that can be given or increases the risk of severe normal tissue injury including osteoradionecrosis, carotid artery rupture, soft tissue necrosis with subsequent sepsis, and even death [9].

Additionally, the Radiation Therapy Oncology Group (RTOG) conducted two Phase II trials of recurrent head and neck SCC with re-irradiation and concurrent chemotherapy [10, 11]. Hydroxyurea/5-fluorouracil with re-irradiation achieved 2- and 5-year survival rates of 15.2 and 3.8%, respectively. Re-irradiation with concurrent cisplatin/paclitaxel achieved 1- and 2-year overall survival rates of 50.2 and 25.9%, respectively, with a median survival time of 12.1 months. However, these treatments came at the cost of increased toxicity. In the concurrent hydroxyurea/5-fluorouracil study, the percentage of patients with grade 3, 4, and 5 acute adverse events was 38, 17.7, and 7.6%, respectively; while in the concurrent cisplatin/paclitaxel group, the percentage of patients with grade 3, 4, and 5 acute adverse events was 49.5, 23.2, and 5.1%, respectively. These historically low survival rates and accompanying substantial toxicity highlight the need for improved strategies.

IBT has been used for several decades for the treatment of both primary and recurrent head and neck tumors. In Puthawala et al.’s study of 220 patients with head and neck recurrences treated with IBT (median dose of 53 Gy) and either chemotherapy with 5-fluorouracil or interstitial hyperthermia as a radiosensitizing/potentiating agent, the 5-year disease free survival rate was 23% and the overall survival rate was 21.7% [12]. Bollet et al. also studied the role IBT played in treating head and neck SCC recurrences without surgical resection. 72 patients were treated with IBT alone to a median dose of 56.5 Gy, while 12 patients were treated to a median dose of 38 Gy in combination with EBRT to 41 Gy. The 1-, 2-, and 5- year overall survival rate was 33, 13, and 1%, respectively, and the 1-, 2-, and 5-year disease-free survival rate was 49, 31, and 0%, respectively. Interestingly, through multivariate analysis, they showed that survival is improved if the time from primary treatment to recurrence is greater than 18 months (*p* < 0.0002) [13]. These results suggest that in well-selected patients, IBT can offer meaningful disease control.

While the efficacy of these IBT^13,^ studies are encouraging, it does not approach that of single modality treatment with surgical resection [6–8] or the outcomes reported in our study. A number of factors may contribute to these disparate results, including patient selection, IBT radiation dosage, and the addition of surgical resection to the treatment plan. This is confirmed by Grimard et al., who noted that surgical salvage with IBT offered improved locoregional control compared to IBT alone [14].

Treatment related complications for salvage surgery and IBT are substantial. In Puthawala et al.’s study 27% of patients suffered from severe (grade 4 or 5) complications of salvage treatment [13], and in Bollet et al.’s study 35% of patients suffered from severe toxic adverse events, with 7% being fatal [14]. In our study, early treatment-related fatalities and carotid blowout did not occur. The large discrepancy between the rate of severe adverse events in these two studies and our study is likely explained by the impact of soft tissue reconstruction, which was utilized in the majority of the patients in this study. Although not the focus of this study, soft tissue reconstruction in this patient population is important to mitigate peri-operative and late radiation-related morbidity.

Attempts to manage recurrent cervical disease with neck dissection and brachytherapy have been ongoing for several decades. Starting in 1975, investigators at Stanford treated a series of patients with postoperative interstitial neck radiation [15]. They chose to use permanent interstitial implants using iodine 125 suture wires. The dose rate was low at 7 cGy per hour. The crude local control in the implant volume in previously irradiated patients was 21/26 (81%). However, only one of the 26 patients remained alive without disease; distant metastases and cervical recurrences outside of the implant volume were the major mode of disease recurrence.

Breen et al.[16] reported a series from Yale of 69 previously irradiated patients who were treated with permanent interstitial brachytherapy. Twenty eight patients underwent therapy for relapsed cervical disease; 24 had disease limited to the neck and four patients also had mucosal disease. The implants were permanent and were performed with either iodine 125 or palladium 103 seed meshes. The median calculated dose was 90 Gy to the tumor surface. The median time to locoregional failure for the 28 patients was 0.6 months. Local control including patients who were treated for mucosal tumors was 28% at 5 years. Patients who had extranodal extension at the time of surgery had significantly worse overall survival. Carotid rupture occurred in two of the 28 patients.

The dose delivery rate of permanent interstitial implants is very low, since the implant source is never removed. This is a disadvantage when treating a tumor such as recurrent squamous carcinoma that has a relatively high proliferation rate and can develop accelerated repopulation of tumor clonigens during therapy. Another problem is that radiation starts immediately albeit at a low dose rate, after the sources are placed instead of several days later as is the case with afterloading implants. The normal tissues and vascular anastomoses of flap reconstructions in the setting of permanent implants do not have any time to heal prior to the start of radiation.

**Fig. 3:**
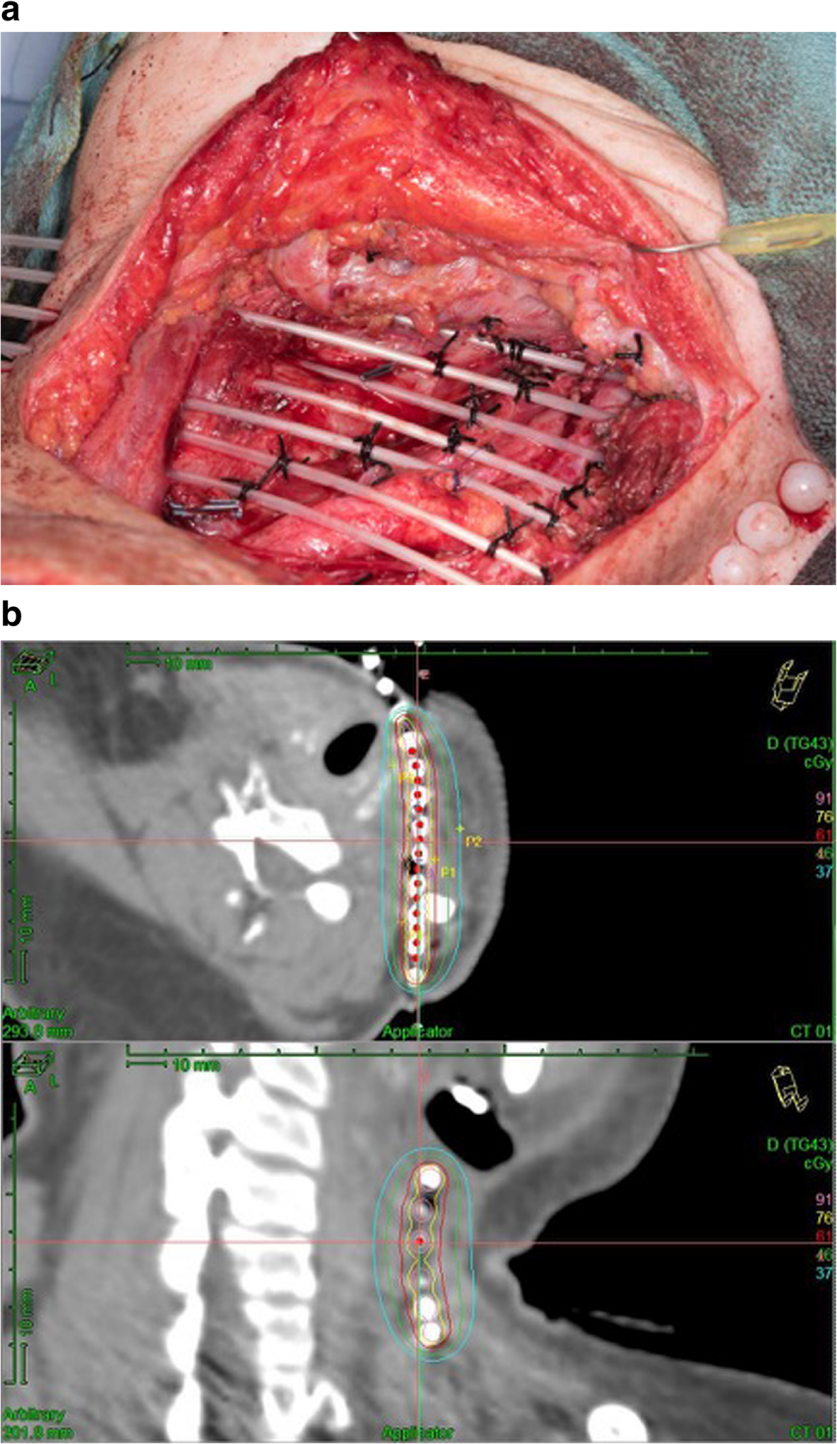
(**a**): Photograph of a brachytherapy implant after neck dissection for cervical recurrence. The catheters are placed in parallel array one cm apart. (**b**): Brachytherapy Dosimetry: The patient was treated to the 61 cGy/hr. line, which is 1 cm wide. The dose declines rapidly with distance, so only a limited volume of the neck is treated

Nutting et al.[17] reported a series of 72 patients treated at the Royal Marsden Hospital between 1979 and 2003 with recurrent cervical adenopathy after previous high dose radiation. The patients had advanced disease, as surgery and brachytherapy was the second curative attempt in 34 patients, third in 24, fourth in 4, and fifth in 10. Stage was rN2 in 58% and rN3 in 17%. Techniques and isotope used were similar to our series (Fig. 3). Interstitial low dose rate brachytherapy was performed after neck dissection using Iridium 192. The implants were initiated between 2 and 8 days after surgery. The final 60 patients in the series had removal of overlying subcutaneous tissue and skin followed by flap reconstruction due to complications seen in the initially treated patients. 60 Gy was delivered to the 85% isodose line using the Paris system. Actuarial disease-specific survival and overall survival was 17 and 23% respectively, at 5 years. For the 60 patients treated with excision, brachytherapy, and flap repair, locoregional control at 2 and 5 years was 37 and 23%, respectively. These 60 patients had a 5 yr. brachytherapy in-field control rate of 66%. The overall major complication rate was 15%, with 3 (4%) patients having a severe hemorrhage, and others having fistula (9%) and wound breakdown (8%).

## Conclusions

Salvage neck dissection with IBT is an aggressive multimodal approach which can achieve a high rate of regional control with acceptable complication rates. At our institution, surgical resection with soft tissue reconstruction and IBT remains our preferred treatment approach for selected patients with isolated/limited neck recurrences following initial treatment that included radiation therapy. The incidence of human papilloma virus-associated oropharyngeal cancer in the United States is increasing, and the treatment strategies for this tend to be radiation-based. The role of surgery and IBT for the treatment of regional recurrences may expand in the future.

## Data Availability

The datasets generated and/or analyzed during the current study are not publicly available due to protected health information but are available from the corresponding author on reasonable request.
